# Acceptability of the 6-PACK falls prevention program: A pre-implementation study in hospitals participating in a cluster randomized controlled trial

**DOI:** 10.1371/journal.pone.0172005

**Published:** 2017-02-15

**Authors:** Anna L. Barker, Renata T. Morello, Darshini R. Ayton, Keith D. Hill, Caroline A. Brand, Patricia M. Livingston, Mari Botti

**Affiliations:** 1 Department of Epidemiology and Preventive Medicine, School of Public Health and Preventive Medicine, Monash University, Melbourne, Victoria, Australia; 2 School of Physiotherapy and Exercise Science, Curtin University, Bentley, Western Australia, Australia; 3 Epworth/Deakin Centre for Clinical Nursing Research, Deakin University, Richmond, Victoria, Australia; 4 School of Nursing and Midwifery, Deakin University, Burwood, Victoria, Australia; University of Ottawa, CANADA

## Abstract

There is limited evidence to support the effectiveness of falls prevention interventions in the acute hospital setting. The 6-PACK falls prevention program includes a fall-risk tool; ‘falls alert’ signs; supervision of patients in the bathroom; ensuring patients’ walking aids are within reach; toileting regimes; low-low beds; and bed/chair alarms. This study explored the acceptability of the 6-PACK program from the perspective of nurses and senior staff prior to its implementation in a randomised controlled trial. A mixed-methods approach was applied involving 24 acute wards from six Australian hospitals**.** Participants were nurses working on participating wards and senior hospital staff including: Nurse Unit Managers; senior physicians; Directors of Nursing; and senior personnel involved in quality and safety or falls prevention. Information on program acceptability (suitability, practicality and benefits) was obtained by surveys, focus groups and interviews. Survey data were analysed descriptively, and focus group and interview data thematically. The survey response rate was 60%. Twelve focus groups (n = 96 nurses) and 24 interviews with senior staff were conducted**.** Falls were identified as a priority patient safety issue and nurses as key players in falls prevention. The 6-PACK program was perceived to offer practical benefits compared to current practice. Nurses agreed fall-risk tools, low-low beds and alert signs were useful for preventing falls (>70%). Views were mixed regarding positioning patients’ walking aid within reach. Practical issues raised included access to equipment; and risk of staff injury with low-low bed use. Bathroom supervision was seen to be beneficial, however not always practical. Views on the program appropriateness and benefits were consistent across nurses and senior staff. Staff perceived the 6-PACK program as suitable, practical and beneficial, and were open to adopting the program. Some practical concerns were raised highlighting issues to be addressed by the implementation plan.

## Introduction

Despite implementation of several activities designed to reduce fall injuries, they remain common in hospitals [1–3]. There is limited high quality evidence to support the effectiveness of prevention interventions in the acute setting [4]. The 6-PACK is a nurse-led falls prevention program designed for acute wards [5]. It includes a fall-risk tool [6] and individualised selection of a ‘falls alert’ sign; bathroom supervision; ensuring patients’ walking aids are within reach; a toileting regime; a low-low bed; and a bed/chair alarm. The program involves nurses assessing their patients’ falls risk each shift and applying a ‘falls alert’ sign and one or more of the remaining 6-PACK interventions to high risk patients. A single-centre study suggests the program is feasible to implement and may reduce fall injuries [7]. A randomised controlled trial (RCT) was conducted to provide robust estimates of effect and generalisability [8]. Prior to the RCT, assessment of the program acceptability was considered important to inform development of a plan to optimise implementation effectiveness [9–11].

Acceptability studies facilitate implementation tailored to local needs and context. There are several components of acceptability including suitability, practicality and benefits. *Suitability*, relates to underlying demand [10] or matching of the program to opportunity (underlying problem) [12]. It can be referred to as ‘appropriateness’ of a program and explores the program’s alignment to needs of patients and likelihood of being used. *Practicality*, relates to the extent to which the program can be implemented efficiently within existing resources [10]. *Benefits*, relate to the program’s potential to achieve intended outcomes—for example, reduce fall-related injuries, and relative advantages above existing care models such as reducing work load for hospital staff. It is also referred to as the ‘potential effectiveness’ [10].

Three prior studies have sought to explore the acceptability of hospital falls prevention programs [11, 13, 14]. The first used a survey to obtain information on barriers and enablers to implementation of a falls prevention guideline from 1,467 nurses in five Singaporean hospitals. High levels of acceptability were reported. However, 25% of nurses reported delivery of the guideline as too time consuming, and 20% that it had limited flexibility with respect to clinical judgement and tailoring for individual patients [11]. The second involved a survey of health care professionals, patients and their relatives (N = 200) at a U.K. general hospital. High levels of acceptability were reported for observation beds, identification bracelets, bed/chair alarms, bed rails and ‘at risk’ labels by the bed [13]. The last study in one sub-acute ward used a survey (N = 12) and focus group (N = 9) of nurses and reported high acceptability of an electronic sensor bed/chair alarm system for patients with cognitive impairment from the perspective of nurses [14]. While the above mentioned studies provide insights, the small sample sizes and single centre designs highlight further studies are required.

This study aimed to explore the acceptability of the 6-PACK program from the perspective of nurses and senior staff prior to implementation of the program as part of a RCT [5]. Specific study objectives were to assess perceived suitability, practicality, and benefits of the program components (fall-risk tool, interventions and integrated care plan) and protocol (nurses to review and update the tool and required interventions each shift). This information would inform development of an implementation plan.

## Materials and methods

### Design

A multi-centre mixed methods pre-implementation study done in accordance with COREQ guidelines (S1 Appendix). This study was part of the 6-PACK project that incorporated a three-year research plan: 1) Studies of current falls prevention practice and moderators (pre-implementation) [15]; 2) A cluster RCT testing 6-PACK effectiveness (S2 Appendix), including economic [16] and program evaluations (implementation); and 3) a longitudinal assessment of sustainability of practice change and outcomes (maintenance).

### Participants and setting

Nurses and senior staff from 24 acute wards (16 medical; 8 surgical) recruited to participate in the RCT were the study participants. Ward recruitment procedures are described in detail elsewhere (S3 Appendix). Nurses were eligible to participate in the survey and/or focus group if they had worked on wards for ≥7.5 hours per week two months prior to survey administration. Staff who did not meet this criteria were excluded as they might have limited knowledge of ward prevention practices and falls. Interviews were conducted with 24 senior staff nominated by the hospital Director of Nursing (DON) and invited by letter from the research team. These included Nurse Unit Managers (NUMs); senior physicians; DONs; and senior personnel involved in quality and safety or falls prevention.

### Nurse survey

A 43-item survey was developed by the research team that included 15 acceptability items (Table 1). The survey was piloted at the hospital that developed and implemented the 6-PACK program to test dissemination approach and comprehension [7]. The length of the survey was the main issue raised by pilot participants, however, it was deemed difficult to further reduce items without losing important content. Formal construct validity of the survey was not undertaken. Survey scores were not intended to be summed or analysed with parametric statistics. Participants rated their agreement to items on a 5-point Likert scale (strongly disagree to strongly agree). The survey was administered to all eligible nurses over two-weeks at each hospital. The researchers described the study purpose, privacy issues and instructions for completion. Completed surveys were placed into a sealed box that was collected by the researcher at the end of the dissemination period.

**Table 1:** Mapping of survey, focus group and interview questions to the acceptability domains.

Survey	Focus group	Interview	Questions/Statements
**Suitability**—*Underlying demand or matching of the program to opportunity and to the care needs of patients*.			
	✓	✓	How does falls prevention compare with other patient safety priorities at your hospital?
✓			Falls are not a problem on my ward so falls prevention programs are not required.
✓			Falls prevention is not a priority on this ward.
	✓	✓	Are falls or fall injuries an issue on your ward/in your hospital?
	✓	✓	What do you see as your role in falls prevention?
✓			Falls prevention is primarily the responsibility of the physiotherapist.
✓			It is not my responsibility to stop patients from falling.
✓			It is my responsibility as the patient's treating nurse to assess their falls risk each shift.
✓			It is my responsibility, to update my patient's falls risk status if a fall and/or change in condition occurs.
	✓	✓	Are you familiar with the six interventions included in the 6-PACK program?
	✓	✓	Would 6-PACK be appropriate for your ward and patients?
		✓	How does the 6-PACK program fit into existing/planned quality and safety programs/other ward/hospital activities?
**Practicality—***Relates to the extent to which the program can be implemented within existing resources and care models*.			
✓			I don't have time to complete a falls risk assessment on all my patients.
✓			Falls risk assessment is a waste of time.
✓			Falls risk assessment tools are a useful way of identifying patients at risk of falling.
✓			A "Falls risk" sign above the bed is a useful way to communicate to staff which patients are at risk of falling.
✓			Low-low beds are an effective way to prevent injuries in patients at risk of falling out of bed.
**Benefits***—Potential to achieve intended outcomes and relative advantage above existing care models*.			
✓			It is my responsibility to implement prevention strategies for patients I identify as high risk
✓			The current falls prevention program is effective at reducing falls on my ward.
✓			Falls risk assessment tools are a useful way of identifying patients at risk of falling.
	✓	✓	What do you think the benefits would be of implementing the 6-PACK program on your ward/in your hospital?
		✓	What outcomes are you seeking from the 6-PACK program and how will you measure these?
	✓	✓	What strategies do you feel are most important for preventing falls?
		✓	What effect, if any, do you feel the 6-PACK project will have on your hospital?
✓			Falls risk assessment tools are better than my own judgment for identifying patients most at risk of falling.

### Focus groups and key informant interviews

Two focus groups and four interviews were scheduled at each hospital. Focus group and interview question guides were developed by researchers experienced in the development, implementation and evaluation of hospital based patient safety programs and informed by relevant literature [15]. Questions related to beliefs about falls; current falls prevention practice; 6-PACK program components; best practice guidelines and key recommendations; and falls reporting practices, and were mapped to the Theoretical Domain Framework (TDF). The TDF includes 12 domains: knowledge; skills; social/professional role and identity; beliefs about capabilities; beliefs about consequences; motivation and goals; memory, attention and decision making processes; environmental context and resources; social influences; emotion; behavioral regulation and nature of the behaviors [17]. Question guides differed slightly between focus groups and interviews. For example, focus groups were with nurses and therefore questions focused on their experiences with falls and preventing falls. Interviews with senior staff included questions that were more operational in nature for example the expected outcomes from the 6-PACK program and how these will be measured. Sessions were led by AB, were 1hr in duration and explored the perceived acceptability of the 6-PACK program (Table 1). Discussions were recorded and transcribed, and transcripts were made available to participants for verification.

### Data analysis

Descriptive statistics were calculated for survey responses using Stata MP v13. Interview and focus group data were analysed by three independent researchers using thematic analysis [18]. Discrepancies were resolved by discussion and consultation with the investigator team as required. All interviews and focus groups were transcribed verbatim and uploaded into Nvivo 11 for data management and analysis. DA coded and recoded transcripts as actions, processes and themes emerged to test applicability and consistency in relation to acceptability of the 6-PACK program. Three rounds of coding were conducted: open, axial and thematic. Deductive theory-driven codes were used to identify overarching acceptability themes based on the survey, FG and interview questions [18]. AB and MB checked the coding framework for the open and axial coding to ensure that coding was consistent. They were also involved in developing the conceptual links and testing for the thematic codes. Quantitative and qualitative data were analysed separately with triangulation at the interpretation stage where findings from each component were considered to determine convergence or divergence [19].

### Ethics

This study was approved by Monash University Human Research Ethics Committee–CF11/0229–2011000072 and relevant hospital ethics committees. Participants were given verbal information about the study and asked to sign consent forms if they were interested in participating.

## Results

### Participants

**Fig 1:**
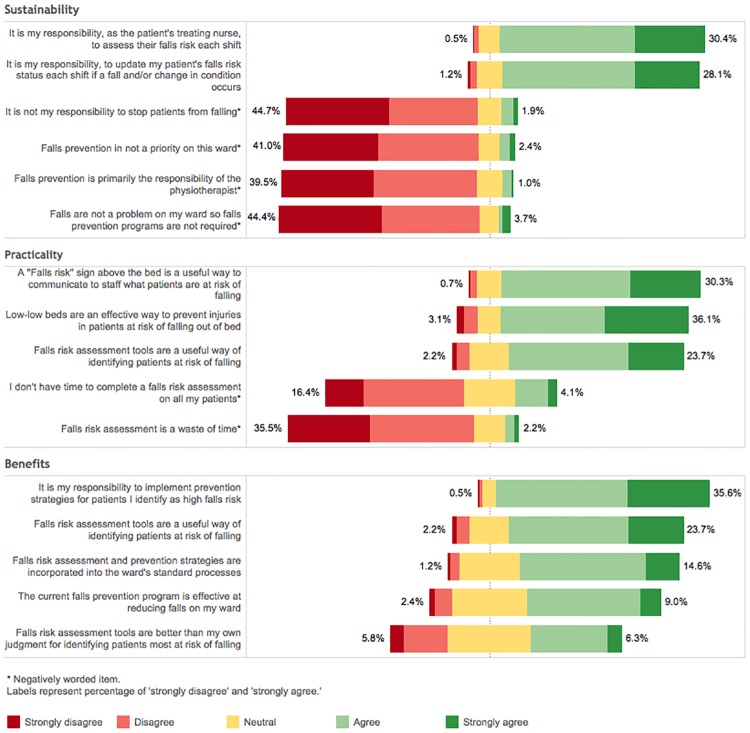
Survey of nurses’ perceived acceptability of key components of the 6-PACK program.

A total of 702 surveys were distributed with 420 returned (60%). Respondents were mostly registered nurses (74%); and medical ward staff (75%) (Table 2). Fig 1 presents the survey results. Twelve focus groups with 96 nurses and 24 interviews with senior staff (SS) were conducted (Table 2).

**Table 2:** Survey, focus group and interview participants.

	Hospital 1	Hospital 2	Hospital 3	Hospital 4	Hospital 5^	Hospital 6^	Total
**Surveys**							
Ward, *n* (%)							
Medical	42 (54.5)	34 (65.4)	87 (77.7)	41 (61.2)	42 (100.0)	70 (100.0)	316 (75.2)
Surgical	35 (45.5)	18 (34.6)	25 (22.3)	26 (38.8)	0 (0.0)	0 (0.0)	104 (24.8)
Qualification, *n* (%)							
RN	68 (88.3)	24 (46.2)	91 (81.3)	39 (58.2)	31 (73.8)	59 (84.3)	312 (74.3)
LPN	3 (3.9)	2 (3.8)	18 (16.1)	4 (6.0)	10 (23.8)	8 (11.4)	45 (10.7)
UAP	1 (1.3)	18 (34.6)	0 (0.0)	18 (26.9)	0 (0.0)	0 (0.0)	37 (8.8)
Not recorded	5 (6.5)	8 (15.4)	3 (2.7)	6 (9.0)	1 (2.4)	3 (4.3)	26 (6.2)
**Focus groups**							
Group 1	8	8	11	5	10	8	50
Group 2	4	9	8	12	9	4	46
Total	12	17	19	17	19	12	96
**Interviews**							
Director of Nursing	1	1	1	1	1	1	6
Nurse Unit Manager	1	1	1	2	1	1	7
Clinical risk coordinator	1	0	0	0	0	0	1
Quality and safety manager	0	0	0	0	1	0	1
Nurse educator	1	1	2	3	0	2	9
Total	4	3	4	6	3	4	24

RN = Registered Nurse; LPN = Licensed practical nurse; UAP = Unlicensed assistive personnel

^No surgical wards at these hospitals participated in the study

Key concepts were identified across the surveys, focus groups and interview responses and mapped to acceptability domains as outlined in Table 3. These were explored in a more in-depth manner below.

**Table 3:** Concepts identified mapped to acceptability domains.

***Suitability***
Falls are the number 1 patient safety problem. Nurses are key players in falls prevention. There is opportunity to improve current falls prevention practice. Risk factors included on 6-PACK fall-risk tool match perceived local risk factors. Alert signs, low-low beds and bathroom supervision were considered matched to local falls problem.
***Practicality***
An integrated care plan is useful and could be used with minimal training. 6-PACK falls risk tool is easy to complete. Time restraints may limit the risk tool and required interventions from being updated regularly. 6-PACK equipment need to be easy to identify, access and well maintained. Completing the risk tool on patients recently admitted can be difficult. Bed/chair alarms can be annoying. There may be privacy issues with using alert signs and bathroom supervision. Bathroom supervision creates a challenge to safely manage other high falls risk patients justify unattended. Bathroom supervision and toileting regimes take time to implement.
***Benefits (and perceived harms)***
An integrated care plan promotes frequent review of patients’ risk status and required interventions. The 6-PACK will bring consistency to falls prevention practice and should reduce falls and fall injuries. Use of a shorter risk tool and fewer interventions will save time. The 6-PACK risk tool provides a useful way to identify patients at risk of falling. ‘Falls alert’ signs increase awareness of patient falls risk amongst staff. Low-low beds reduce injuries from falls. Bathroom supervision prevents bathroom falls. Toileting regimes may exacerbate continence issues. Positioning patients’ walking aids in reach may increase falls. Staff and patients may incur injuries with low-low bed use. Bed/chair alarms may not be effective if used in isolation.

### Matching of program to opportunity

The 6-PACK program was perceived to be suitable with high levels of demand for a new falls prevention approach. Survey data indicated falls remained a problem—84% of nurses disagreed with the statement *‘Falls are not a problem on my ward so falls prevention programs are not required’*. Staff believed that falls remained their *“leading incident”*. Current falls prevention activities were perceived to have limited effectiveness.

Two patients have died…falls are such an important issue for our patients

*(SS1, Hospital (H)4)*.

I think there’s a lot more we can do in terms of falls prevention

(Nurse, H4)

Staff highlighted current falls prevention practice was inconsistent and that the 6-PACK program could address this.

We don’t always implement things in a structured manner. It [6-PACK program] gives us a really structured way of implementing

*(SS1, H5)*.

### Integrated care plan with daily nurse review

The program was considered suitable. Nurses agreed it was their responsibility to assess patients’ fall risk status each shift (86%) and to implement interventions for high risk patients (90%). Inclusion of the fall-risk tool and interventions on the care plan was perceived to be a practical, suitable and a beneficial improvement on current practice as it promoted more frequent review of patients’ risk status.

[The care plan is] really good…If I didn’t know that patient and I came to care for them, I would know straightaway I had to check their falls risk. You are more alert to making sure strategies are in place. Nurses will like it

(SS2, H5)

The 6-PACK care plan was identified as practical to use in the busy ward environment. Nurses felt check boxes would save time *“because it only takes 10 seconds”* and were easy to use. Some concerns were raised whether the review and updating of the care plan would occur consistently.

### Fall-risk tool

Nurses believed fall-risk tools were useful for identifying patients at risk of falling (73%). Nurse and senior staff felt the 6-PACK tool was suitable and more practical than current tools. It was recognised as being shorter and simpler, with appropriate risk factors and use of a two rather than three-level risk status (i.e. high or low v. low, medium or high).

*[Our tool is] four pages long. It is not meaningful to staff because they just see it as cumbersome*.

(SS1, H5)

*[The current tool] doesn’t identify very well people at risk of falling*.

(Nurse, H1)

Practical barriers to completion of the tool for patients recently admitted were identified.

*If the patient has just been transferred I have to observe them before I can complete the tool properly. If there's no family around, you go through all the files, it takes more than 10 minutes to do properly*.

(Nurse, H5)

Despite acknowledging completing fall-risk tools take time, 80% of nurses disagreed that they were a waste of time.

### Alert signs

Nurses reported signs were already used to some extent on wards highlighting suitability. Nurses reported signs were useful at communicating patients’ falls risk status to the care team (73%) and had the potential to decrease falls.

*I find signs effective…the moment I enter the room and I see it I’ll be aware that the patient is high falls risk, I’ll keep an eye on them*.

(Nurse, H5)

They were considered particularly beneficial when attending patients not known by staff.

A practical barrier to sign use was ease of access.

*We do have signs … they are just not in the room and not accessible*.

(Nurse, H4)

### Bathroom supervision

Supervising patients in the bathroom was considered suitable and beneficial based on knowledge that many falls occur in the bathroom amongst unsupervised patients. Currently, it was used variably.

*I think presence in the bathroom is really important. It’s something that can get missed. You find patients left in the bathroom that you know shouldn’t have been*.

(SS1, H3)

Practical barriers to bathroom supervision were identified. Firstly, bathroom supervision of one patient meant that other patients are left unattended.

*If you’re in the bathroom with someone and another patient buzzed, you get a phone call or page it’s really challenging to stay in the bathroom*.

(Nurse, H1)

The second barrier identified was privacy. Some nurses believed bathroom supervision was uncomfortable for patients and nurses.

*You want to just say, ‘I’m just out here and I’ll check in’, and then you hear crash, bang. You try to give them that little bit of dignity that they’ve got left to go to the toilet in peace*.

(Nurse, H1)

The third barrier identified was limited time.

*Sometimes we don’t have time to supervise them on the toilet*…*we’ve got a lot of things to do*.

(Nurse, H1)

The use of ‘partner’ nursing was identified as a strategy to promote bathroom supervision.

*Working with a partner, you know you’ve got each other’s patients if something comes up that you can't attend*.

(Nurse, H1)

### Patients’ walking aids within reach

There were mixed beliefs regarding the benefits of positioning patients’ walking aid within reach. Some acknowledged if a patient is going to get up it is best to *“give them something to hold on to”* whilst others felt it was *“dangerous having the walking aid near the patient”* as *“patients can trip on it”*. Concerns were also raised about the suitability and benefits of this intervention for people with cognitive impairment.

*If they’ve got dementia they’re just as likely to fall over with the walking aid as without*.

(Nurse, H3)

### Toileting regimes

Nurses and senior staff expressed contrasting views regarding toileting regimes. Senior staff believed they were useful while nurses reported other interventions such as the use of bed pans to be more practical, allowing them to continue to supervise other patients.

*We encourage all our falls risk patients are second-hourly toileted*.

(SS2, Hospital 3)

*If I took them [to the toilet] every two hours I'm going to make their bladder worse*.

(Nurse, H5)

*Sometimes we just have to use a bedpan, so at least we don’t have to take them to the toilet and stay with them*.

(Nurse, H2)

There were different opinions as to whether toileting regimes are easier to implement during the day or at night.

*At night, when you’ve got 8–10 patients…you can’t toilet someone on a schedule when you’ve got that many to worry about*.

(Nurse, H4)

*[Toileting schedules are] easy to do on night duty because you have your regular rounds, but during the day I find it disruptive and virtually impossible*.

(Nurse, H1)

### Low-low beds

Nurses agreed low-low beds were an effective intervention to minimise injuries in patients that fall getting out of bed (80%). Staff highlighted “*even if patients were to fall*, *the height reduces the impact and injuries”*.

Practical barriers to the use of low-low beds were identified including accessibility.

We know these patients are fallers but we’ve only got so many beds…So it’s always a battle, which I think is a bit of a barrier. How do I get a bed? How do I hire one?

(SS3, H3)

Concerns were also raised regarding their potential to increase staff and patient injury.

*I’ve seen a nurse trip over [the low-low bed] and hit her head on the bedside table*.

(Nurse, H3)

*Low-low beds are helpful but are hard for staff. Once the patient is on the floor it's hard to lift them back to bed*.

(Nurse, H6)

*They rolled out and hit their head against the bedside locker*.

(Nurse, H1)

Issues regarding the identification, ease of use and practicality of the beds were also raised.

*They are not marked. You don’t know which ones are the low-low beds*.

(Nurse, H3)

*You can’t use them for transport. You can’t put folders or air mattresses on them*.

(Nurse, H1)

### Bed/chair alarm

Nurses had mixed beliefs about the benefits of alarms. Some believed they were only useful if used in conjunction with other interventions.

*You’ve got to use alarms in conjunction with the patient being close to the nurses’ station. It’s no good having them right up the other end where you hear the alarm and by the time you’ve got there the patient’s fallen over*.

(SS4, H3)

Practical barriers to alarm use included a perception they were annoying and therefore ignored, that they increase workload and are often broken.

*After a while I’d just get sick of it and just ignore it*.

(SS1, H6)

*I think they're good however can be temperamental…they're not long-lasting*.

(SS1, H5)

*They don't help your workload because every five minutes they go off and you have to respond even if you're busy elsewhere*.

(Nurse, H6)

### Overall impressions

Staff identified the 6-PACK program promoted more *“standardised”*, *“streamlined” and “consistent”* use of interventions than was undertaken in current practice. Staff believed the program offered potential to reduce falls and injuries highlighting potential effectiveness.

*You’d have to see some reduction in falls and falls related injuries after implementing the 6-PACK*.

(Nurse, H1)

## Discussion

Nurses have a key role in falls prevention. This study extends prior studies [11, 13, 14] by seeking information not only from nurses but also senior staff providing a detailed understanding of falls prevention practice in acute hospitals. This is the first multi-centre, mixed methods study of the acceptability of many commonly used falls prevention interventions. Staff perceived the 6-PACK program is suitable and for the most part practical and beneficial. Some practicality concerns were raised highlighting targets to be addressed by the implementation plan.

Staff highlighted need for a new falls prevention approach. Falls are perceived as prevalent and deleterious, and existing prevention practices have numerous limitations. The 6-PACK was perceived to offer advantages over existing practice. Staff believed it was practical—a short, simple risk tool, integrated care plan, and focus on only a few interventions—and was appropriate for the needs of their patients—risk factors on the tool were considered relevant and the interventions were considered useful in local context. Others have also identified a need for simple programs with nurses reporting *‘too many must do's are daunting’* [20] and that integration into exiting work practices facilitates practice change [21]. Staff agreed with the program focus on nurses and identified falls as a nursing sensitive outcome, consistent with prior literature [22]. The program was considered easy to integrate into existing care and able to be used with minimal training.

Staff perceived many benefits to both themselves and patients with implementation of the 6-PACK program. Benefits included more frequent review of patients’ risk status, a more consistent falls prevention approach across staff and reduced time spent on documentation. Signs were considered an effective means of communicating falls risk amongst staff that would increase use of interventions, consistent with U.K. studies [13]. Low-low beds were believed to be effective for reducing injuries from bed falls, and bathroom supervision an effective way to prevent bathroom falls.

The perceived suitability and benefits of the 6-PACK program suggests a high likelihood of the program being used by nurses and use supported by senior staff. A perceived lack of time, access to equipment, and patient privacy issues may compromise the use of the program. Time constraints and access to equipment have been identified by prior falls prevention studies as factors limiting uptake [11, 20]. The implementation plan must ensure there is appropriate access to signs, low-low beds and alarms. This includes adequate provision of equipment to meet patient demand, storage of equipment in patient rooms, and clear labelling so it is easily identifiable. Access to equipment was identified in a large study of U.S hospitals as a factor reducing opportunity to provide safe care [23]. Staff time, workload and resource constraints are the most commonly reported barriers to implementing nursing practice guidelines [21]. Education sessions should address privacy issues with bathroom supervision. Use of dignity gowns and avoiding direct eye contact while patients are voiding could be promoted.

Consistent with others [13, 14], we identified that nurses perceived bed/chair alarms as a useful way to prevent patient falls. However, nurses raised alarms were often not in working order, are less effective when used in isolation or when too many are used at once. This highlights the need for regular maintenance audits, education that promotes the use of alarms in combination with other interventions and guidelines about prioritising use to only 1–3 patients on a ward at a time.

Some staff also identified risks associated with the use of the 6-PACK. ‘Partner’ nursing was identified as a strategy to mitigate the risk of leaving patients unattended when providing bathroom supervision. While there is evidence that increased levels of nursing (higher nurse-patient ratios) are associated with fewer falls [24, 25], there is an absence of evidence regarding associations between ‘partner’ nursing and falls. A Canadian study reported nurses were concerned about the fall hazard created by keeping walking aids within reach especially when bedside space is limited [20]. Education should include recommendations on ensuring the bedside area is clear of clutter to minimise patient injury. Education should also recommend the patient’s bed is raised to an appropriate height during transfers and care activities.

In this study, perspectives from both nurses and senior staff provided a detailed understanding of falls prevention in acute hospitals, contributing to knowledge of how to enhance implementation fidelity of programs. Focus groups and interviews provided more comprehensive information on beliefs about effectively preventing falls, compared to survey only methods [11]. This study was conducted as part of the 6-PACK RCT [8] which introduced bias as participants were recruited from hospitals that had volunteered to participate in the RCT.

The 6-PACK program is nurse-led and therefore our focus was to seek the perspectives of nurses. Doctors, pharmacists and allied health professionals are also important falls prevention stakeholders and may have different perspectives. Further research to explore this is warranted. Patient and family perspectives would also be important to consider. Additional publications present the findings of the other pre-implementation studies conducted as part of the 6-PACK project [8] including a profile of safety climate, review of current falls prevention practice, in-hospital fall epidemiology, and investigation of implementation barriers and enablers.

## Conclusions

While the 6-PACK program remained fixed in the RCT, implementation was tailored to local context to optimise implementation and effects. This study confirmed the acceptability of the 6-PACK program to nurses who are the program end-users and senior staff who are executive sponsors. Staff believed the program to be a suitable, practical and beneficial way to assist them to reduce falls. Information obtained from this study was incorporated with that from other pre-implementation studies to develop the implementation RCT plan.

## Supporting information

S1 Appendix COREQ checklist. (DOCX)

S2 Appendix 6-PACK programme to decrease fall injuries in acute hospitals: Cluster randomised controlled trial (published article). (PDF)

S3 Appendix Development of an implementation plan for the 6-PACK falls prevention programme as part of a randomised controlled trial: Protocol for a series of preimplementation studies (published article). (PDF)
