# Supplementary material for: Acceptability of the 6-PACK falls prevention program: A pre-implementation study in hospitals participating in a cluster randomized controlled trial
Source: PLoS One. 2017 Feb 15;12(2):e0172005. doi: 10.1371/journal.pone.0172005 (PMC5310900; doi:10.1371/journal.pone.0172005)
Supplement: S1 Appendix — (DOCX) [file pone.0172005.s001.docx]

**Acceptability of the 6-PACK falls prevention program: A pre-implementation study in hospitals participating in a cluster randomised controlled trial – COREQ checklist**

|  | **Item** | **Guide questions/description** | **Response** |
| --- | --- | --- | --- |
| ***Domain 1: Research team and reflexivity*** | | | |
| Personal Characteristics | Interviewer/facilitator | Which author/s conducted the interview or focus group? | Anna Barker ran all of the interviews and focus groups.  Renata Morello and Mari Botti also assisted with data collection. |
|  | Credentials | What were the researcher's credentials? E.g. PhD, MD | Anna Barker: PhD, MPhys (Geriatrics), BPhys  Renata Morello: MPH, BPhys  Mari Botti: RN, BA, PhD |
|  | Occupation | What was their occupation at the time of the study? | Anna Barker: Senior Research Fellow, Falls and Bone Health Team, Monash University; Physiotherapist  Renata Morello: Research Manager, Falls and Bone Health Team, Monash University; Physiotherapist  Mari Botti: Professor, Deakin University |
|  | Gender | Was the researcher male or female? | Female |
|  | Experience and training | What experience or training did the researcher have? | All the researchers are experienced researchers in the health services setting. They all have experience with quantitative and qualitative methods |
|  | Relationship established | Was a relationship established prior to study commencement? | No |
| Relationship with participants | Participant knowledge of the interviewer | What did the participants know about the researcher? e.g. personal goals, reasons for doing the research | Rationale for the research was outlined to participants in the information sheets and at the beginning of the data collection encounter. The participants understood that this data collection was to inform the implementation of the 6-PACK RCT. |
|  | Interviewer characteristics | What characteristics were reported about the interviewer/facilitator? e.g. Bias, assumptions, reasons and interests in the research topic | None – the interviewer/facilitator did not have any biases or assumptions. Anna Barker did conduct an observational study on the 6-PACK program however she was not involved in the development of the program and did not have a vested interest in the outcome of the RCT. |
| ***Domain 2: Study design*** | | | |
| Theoretical framework | Methodological orientation and theory | What methodological orientation was stated to underpin the study? e.g. grounded theory, discourse analysis, ethnography, phenomenology, content analysis | This study is a concurrent mixed methods study. |
| Participant selection | Sampling | How were participants selected? e.g. purposive, convenience, consecutive, snowball | Purposive and convenience sampling were utilized |
|  | Method of approach | How were participants approached? e.g. face-to-face, telephone, mail, email | Senior staff received a letter of invitation to participate in the interview.  Nursing staff were verbally invited to participate in the focus group at handover |
|  | Sample size | How many participants were in the study? | 96 nurses participated in the focus group  24 senior staff participated in interviews  420 nurses completed the survey |
|  | Non participation | How many people refused to participate or dropped out? Reasons? | 702 surveys were distributed – response rate of 60%  Response rate for focus groups is unknown.  All those invited to participate in an interview consented. |
| Setting | Setting of data collection | Where was the data collected? e.g. home, clinic, workplace | At each of the 6-PACK study hospitals |
|  | Presence of non-participants | Was anyone else present besides the participants and researchers? | No |
|  | Description of sample | What are the important characteristics of the sample? e.g. demographic data, date | Personal demographic information was not collected (e.g. age gender). Demographic information collected and reported included qualifications, primary nursing role, and length of time working at the hospital. |
| Data collection | Focus group guide and interview guide | Were questions, prompts, guides provided by the authors? Was it pilot tested? | Participants were not provided with the interview/focus group questions prior to the data collection encounter. The interview/focus group was not pilot tested |
|  | Repeat interviews | Were repeat interviews carried out? If yes, how many? | No repeat interviews were conducted |
|  | Audio/visual recording | Did the research use audio or visual recording to collect the data? | All interviews and focus groups were audio recorded |
|  | Field notes | Were field notes made during and/or after the interview or focus group? | No |
|  | Duration | What was the duration of the interviews or focus group? | Interviews went for approximately 1 hour and focus groups were approximately 1.5 hours |
|  | Data saturation | Was data saturation discussed? | Data saturation was reached however the sampling was purposive in that two focus groups were run at each hospital and interviews were conducted with senior staff in key roles at each hospital. Therefore the data collection aimed to be representative of the hospital in the study (nurse perspectives and senior staff perspectives). |
|  | Transcripts returned | Were transcripts returned to participants for comment and/or correction? | No |
| ***Domain 3 Analysis and findings*** | | | |
| Data analysis | Number of data coders | How many data coders coded the data? | Three |
|  | Description of the coding tree | Did authors provide a description of the coding tree? | The coding tree in terms of major and minor themes is presented in table 2. |
|  | Derivation of themes | Were themes identified in advance or derived from the data? | Acceptability domains (suitability, practicality and benefits) were identified from the literature and deductively applied to the data. Inductively derived themes were identified and discussed in the context of the deductive major themes. |
|  | Software | What software, if applicable, was used to manage the data? | Nvivo |
|  | Participant checking | Did participants provide feedback on the findings? | No |
| Reporting | Quotations presented | Were participant quotations presented to illustrate the themes / findings? Was each quotation identified? e.g. participant number | Yes – quotes have been provided to illustrate themes. The participant role (nurse or senior staff) and hospital identifier have been included with the quotes. |
|  | Data and findings consistent | Was there consistency between the data presented and the findings? | Yes – the study aimed to gain insight into nurse and senior staff perceptions of the acceptability of the 6 PACK program. The results are presented using the major themes of suitability, practicality and benefits (identified from the literature) with emerging minor themes described for each major theme. |
|  | Clarity of major themes | Were major themes clearly presented in the findings? | The major themes were deductively derived from the academic literature. |
|  | Clarity of minor themes | Is there a description of diverse cases or discussion of minor themes? | The minor themes were inductively derived and embedded within the major themes. |
