# Supplementary material for: Acceptability of the 6-PACK falls prevention program: A pre-implementation study in hospitals participating in a cluster randomized controlled trial
Source: PLoS One. 2017 Feb 15;12(2):e0172005. doi: 10.1371/journal.pone.0172005 (PMC5310900; doi:10.1371/journal.pone.0172005)
Supplement: S3 Appendix — (PDF) [file pone.0172005.s003.pdf]

Downloaded from http://injuryprevention.bmj.com/ on March 23, 2016 - Published by group.bmj.com
IP Online First, published on March 1, 2016 as 10.1136/injuryprev-2015-041915

Development of an implementation plan for the 6-PACK falls prevention programme as part of a randomised controlled trial: protocol for a series of preimplementation studies

Anna L Barker,[1] Renata T Morello,[1] Darshini R Ayton,[1] Keith D Hill,[2] Fiona S Landgren,[3] Caroline A Brand[1]

[1]Department of Epidemiology and Preventive Medicine, School of Public Health and Preventive Medicine, Monash University, Melbourne, Australia
[2]School of Physiotherapy and Exercise Science, Curtin University, Bentley, Australia
[3]Project Health, Melbourne, Australia

Correspondence to Professor Anna Barker, Monash University, Department of Epidemiology and Preventive Medicine, The Alfred Centre, 99 Commercial Road, Melbourne, VIC 3004, Australia; anna.barker@monash.edu

Received 26 November 2015
Accepted 27 November 2015

ABSTRACT

Background Inhospital falls cause morbidity, staff burden and increased healthcare costs. It is unclear if the persistent problem of inhospital falls is due to the use of ineffective interventions or their suboptimal implementation. The 6-PACK programme appears to reduce fall injuries and a randomised controlled trial (RCT) was undertaken to confirm effects. This paper describes the protocol for the preimplementation studies that aimed to identify moderators of the effective use of the 6-PACK programme to inform the development of an implementation plan to be applied in the RCT.

Methods The 6-PACK project included five preimplementation studies: (1) a profile of safety climate; (2) review of current falls prevention practice; (3) epidemiology of inhospital falls; (4) acceptability of the 6-PACK programme; and (5) barriers and enablers to implementation of the 6-PACK programme. The Theoretical Domain Framework that includes 12 behaviour change domains informed the design of these studies that involved 540 staff and 8877 patients from 24 wards from six Australian hospitals. Qualitative and quantitative methods were applied with data collected via: structured bedside observation; daily nurse unit manager verbal report of falls; audit of medical records, incident reporting and hospital administrative data; surveys of ward nurses; focus groups with ward nurses; and key informant interviews with senior staff.

Discussion Information on contextual, system, intervention, patient and provider level factors is critical to the development of an implementation plan. Information gained from these studies was used to develop a plan applied in the RCT that addressed the barriers and harnessed enablers.

Trial registration number The RCT is registered with the Australian New Zealand Clinical Trials Registry, number ACTRN12611000332921.

BACKGROUND

Patient falls remain the most common cause of harm in acute hospitals worldwide. This is despite the substantial investment in the development and dissemination of best practice guidelines,[1–3] the adoption of inhospital falls as a quality indicator[4 5] and the implementation of several programmes[6 7] and initiatives aimed at reducing falls. However, there is evidence that there remains considerable scope to improve falls prevention practice in acute hospitals.[8 9] Inhospital falls remain the most commonly reported safety incident in National Health Service (NHS) hospitals[10] with more than 240 000 falls each year in England and Wales.[11] Additional hospital stay costs are estimated to range between £3332 and £27 468 per fall, depending on the injury.[12] This is consistent with international studies which report rates of major inhospital fall injuries have increased over the last decade in Denmark[13] and that rates of injurious falls remain unchanged in the USA.[14] In Australia, an inhospital fall is associated with an increase in hospital stay of 8 days, and $A6669 additional hospital costs.[15]

One factor that may partially explain the persistent problem of falls in acute hospitals is suboptimal adoption of falls prevention practices. Hospital falls prevention programmes may be vulnerable to suboptimal implementation due to their complexity—they have many interacting components in contrast to simple interventions such as infection control through hand hygiene strategies.[16] The Medical Research Council's framework for the evaluation of complex interventions defines a complex intervention as "built up from a number of components, which may act independently and interdependently".[17] Inhospital falls result from a myriad of interactions between physiological, behavioural and environmental factors and their prevention is dependent on the knowledge, skills and behaviours of clinical staff, and the cultural and physical environment, availability of resources, and the policies, incentives and penalties that govern and drive practice.

Several factors can influence the implementation and integration of interventions into clinical practice. These include environmental and contextual issues; staff knowledge, beliefs and attitudes; organisational culture and climate; staff workloads; and access to appropriate equipment and resources. Some factors act as an implementation enabler and others a barrier. An in-depth understanding of the barriers and enablers surrounding the use of a particular intervention can inform the development of an implementation plan. Once delineated, in theory, the barriers can be dealt with and overcome, and enablers harnessed to optimise implementation and effectiveness of the intervention.[18]

The 6-PACK programme is a targeted nurse-led multifactorial falls prevention programme designed specifically for acute wards. It includes a short fall-risk tool[19] and six interventions (figure 1).[20] A 9 year observational study conducted in one hospital indicated the programme could be

To cite: Barker AL, Morello RT, Ayton DR, *et al*. *Inj Prev* Published Online First: [*please include* Day Month Year] doi:10.1136/injuryprev-2015-041915

Copyright Article author (or their employer) 2016. Produced by BMJ Publishing Group Ltd under licence.

Downloaded from http://injuryprevention.bmj.com/ on March 23, 2016 - Published by group.bmj.com

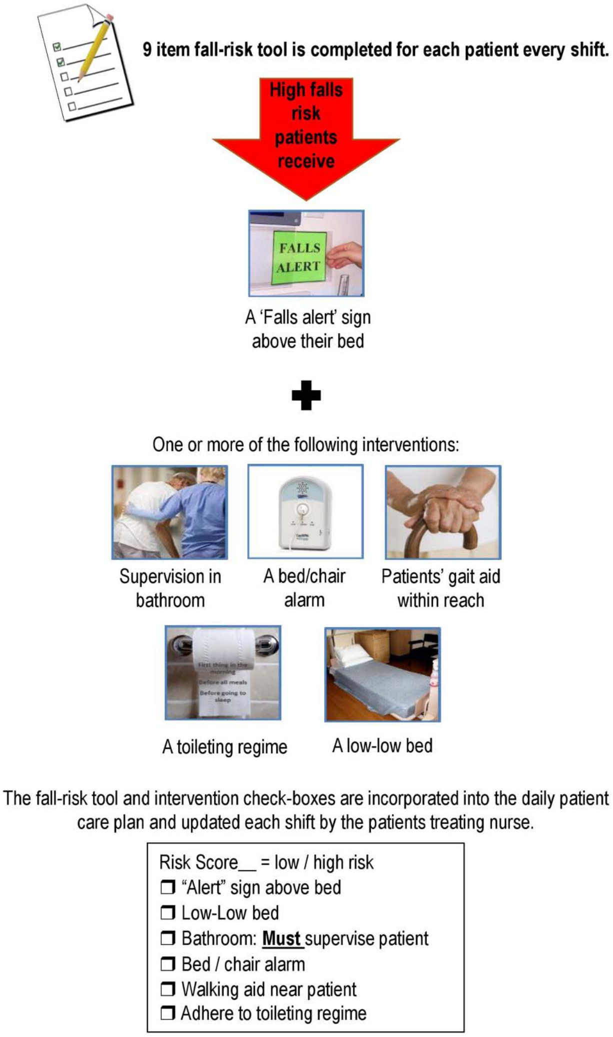

Figure 1 The 6-PACK programme.

implemented in acute wards and may reduce fall injuries.[21] A large-scale randomised controlled trial (RCT) was undertaken to provide robust estimates of effect and information on generalisability to settings external to where the programme was developed.[20] The trial was part of the broader 6-PACK project which included preimplementation, programme and economic[22] evaluation studies. This paper provides an overview of the methods applied for five preimplementation studies undertaken as part of the 6-PACK project.

Aims

The overall aim of the five preimplementation studies was to obtain information on moderators of the effective use of the 6-PACK programme to inform the development of a tailored implementation plan that could be applied in the RCT. To achieve this, the following objectives were established:

1. To understand the profile of safety climate in participating wards.
2. To identify current falls prevention practice on participating wards and specific behaviours required to change to effectively implement the 6-PACK.
3. To describe the epidemiology (patterns, causes and effects) of falls in participating wards.
4. To examine the acceptability (perceived suitability, practicality and benefits) of the 6-PACK programme from the perspective of nurses and senior staff; and
5. To describe nurse and senior staff perceived barriers to and enablers of the programme implementation.

Guiding frameworks

The theoretical framework guiding the design of the preimplementation studies is the Theoretical Domain Framework (TDF). The TDF has been widely used to identify implementation challenges and design behaviour change and implementation strategies for complex health interventions in a variety of contexts. The TDF includes 12 domains: knowledge; skills; social/professional role and identity; beliefs about capabilities; beliefs about consequences; motivation and goals; memory, attention and decision-making processes; environmental context and resources; social influences; emotion; behavioural regulation and nature of the behaviours.[23] The theoretical domains are proposed to be potential moderators of behaviour change (except for 'nature of the behaviours', which relates to the essential characteristics of the behaviour of interest rather than possible mediating mechanisms or influences on behaviour).

METHODS

Design and timelines

This study was part of the 6-PACK project that incorporated a 3-year research plan: (1) Studies of current falls prevention practice and moderators (preimplementation); (2) A cluster RCT testing 6-PACK effectiveness,[20] including economic[22] and programme evaluations (implementation); and (3) An assessment of sustainability of practice change and outcomes (maintenance) (figure 2).

A programme logic model was developed for the project to articulate the causal links between elements of the programme and its intended outcomes (figure 3). It displays the '*logic*' or assumptions on which programme activities are based by sequentially linking inputs, activities and outputs to outcomes (intended and unintended), providing a visual map of what a project will do and how it will be achieved.[24] It informs the development of the implementation plan.

The development of the programme logic was guided by the experiences of implementing and sustaining the 6-PACK programme at The Northern Hospital—the hospital where the programme was developed, implemented and initial evaluation conducted.[21] Current evidence, theory and the opinions of people experienced in the implementation and evaluation of hospital based patient safety programmes informed the design of the programme logic.

Qualitative and quantitative methods were applied to investigate the 6-PACK programme from several perspectives. Each of the five objectives was achieved by undertaking five interlinked preimplementation studies that were conducted concurrently (figure 4). Data were collected over a 6 month period in 2011–2012.

Participants and setting

Information was collected from 540 staff and 8877 patients from 8 surgical and 16 medical wards across six hospitals in Australia recruited to participate in the 6-PACK RCT. Ward recruitment procedures were described in detail elsewhere.[20] In brief, six public hospitals ranging in size from moderate (200–500 beds) to large (>500 beds) in metropolitan and regional Victoria, and metropolitan New South Wales, Australia agreed to participate in the RCT. Each hospital identified wards where the average patient length of stay was less than 10 days; where falls commonly occurred, and that had low levels of use of the falls prevention interventions being tested in the 6-PACK

Barker AL, *et al*. *Inj Prev* 2015;**0**:1–7. doi:10.1136/injuryprev-2015-041915

Downloaded from http://injuryprevention.bmj.com/ on March 23, 2016 - Published by group.bmj.com

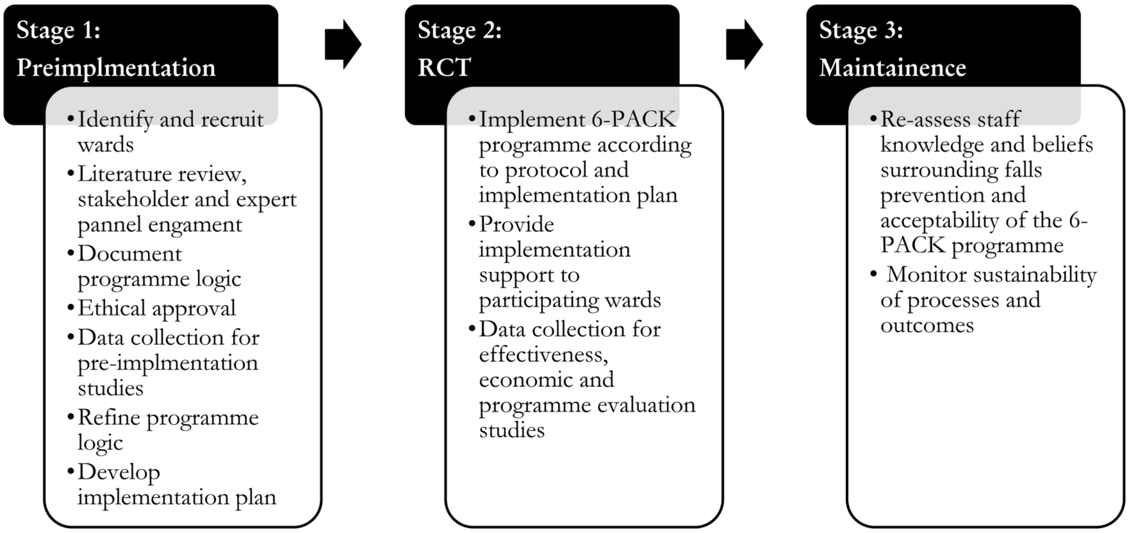

Figure 2 Temporal summary of 6-PACK project activities. RCT, randomised controlled trial.

project. The preimplementation studies were conducted in the same wards as those recruited for the RCT, prior to commencing the RCT.

A waiver of individual patient consent was provided by hospital ethics committees enabling all patients admitted to the study wards during the preimplementation study period to be included as study participants. There were no patient-level inclusion or exclusion criteria.

Nurses were eligible to participate in the survey and/or focus group if they had worked on the participating wards for more than 7.5 h per week in the 2 months prior to administration of the survey. Staff who did not meet the above criteria were excluded from this study because they might have had limited knowledge of, or exposure to, the ward (and hospital) falls prevention practices and falls. Key informant interviews were conducted with 24 senior staff from participating hospitals. A range of managers were nominated by the Director of Nursing at each hospital and were invited to participate by a letter from the research team. These staff included a mix of nurse unit managers (NUMs); senior physicians; directors of nursing and clinical services; and senior personnel involved in quality, safety, risk management and falls prevention.

Data collection

Data were collected via: (1) structured bedside observation; (2) daily NUM verbal report of falls; (3) audit of medical records, incident reporting and hospital administrative data; (4) survey

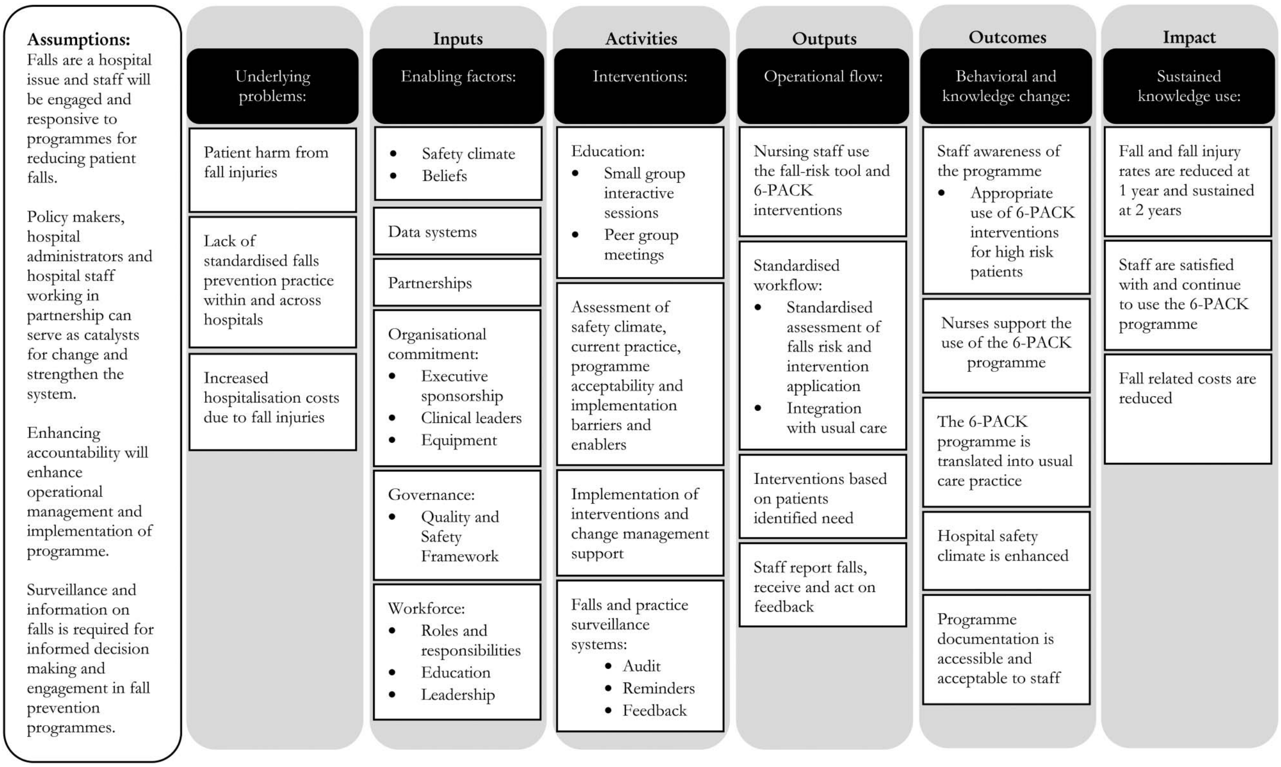

Figure 3 Logic model for the 6-PACK project.

Downloaded from http://injuryprevention.bmj.com/ on March 23, 2016 - Published by group.bmj.com

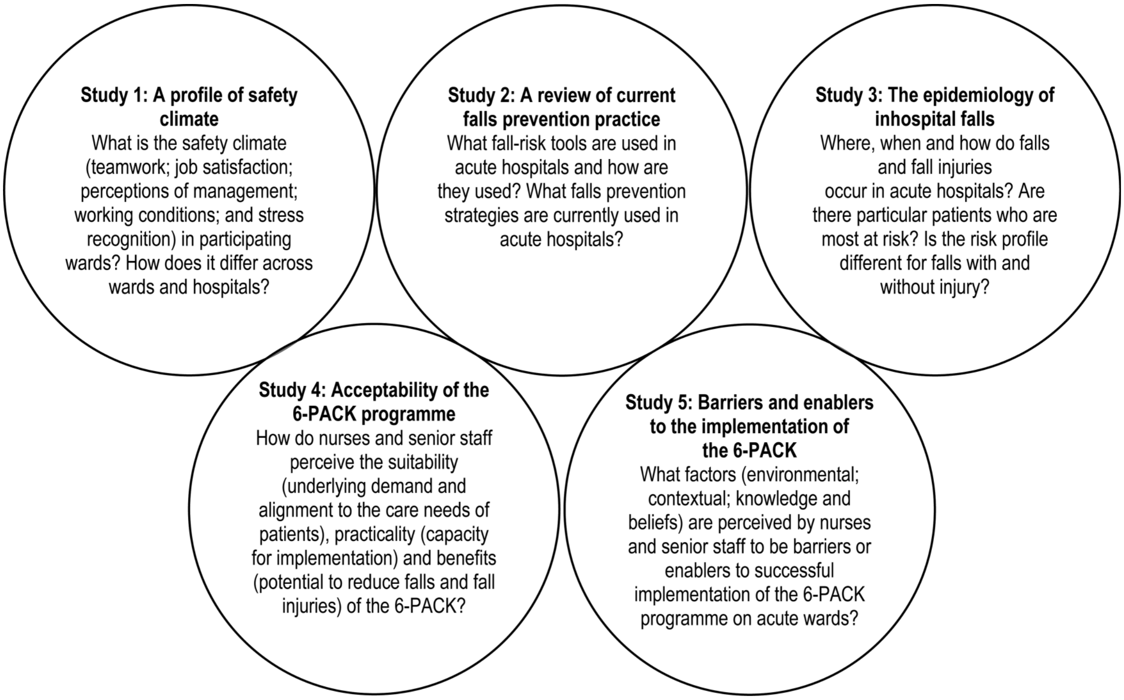

Figure 4 6-PACK preimplementation studies.

of ward nurses; (5) focus groups with ward nurses; and (6) key informant interviews with senior staff (table 1).

Structured observation of patient's bedside

Data on falls prevention strategies applied for all patients admitted to the study wards were recorded by structured bedside observation by trained assessors using a standardised tool for each day of the study. Information on environmental strategies (eg, ensuring that the room had adequate lighting and was free of cluster); use of falls prevention equipment (eg, low-low beds and bed/chair alarms); and surveillance and identification strategies (eg, positioning in a high visibility area, falls alert bracelet on the patient; alert signs above the bed) were recorded.

NUM verbal report

Information on fall events was prospectively collected via daily verbal reports from the ward NUM. The NUM was questioned by a trained assessor each day about falls that were known to have occurred within the previous 24 h on each study ward. Information relating to: the date and time of the fall; location of the fall (bathroom, bedroom, bedside, not on the ward, other ward area); fall mechanism (dressing, moving in/out of bed/chair, moving on/off toilet, rolled out of standard/low-low bed, showering, walking, walking to/from toilet); whether equipment contributed to the patient falling (eg, gait aid, chair, bed); whether the fall was witnessed (person standing within arm's length of patient when falling) or unwitnessed; whether the patient was confused at the time of the fall; fall injuries (eg, bruise, skin tear, fracture) and any postfall strategies that were implemented, was collected.

Audit of medical records, incident reporting and hospital administrative data

Information on required falls prevention strategies, risk tool scores, and fall information documented in the medical record for all patients admitted to the study wards was recorded by daily medical record audit by trained assessors using a standardised tool. Data elements related to falls prevention strategies

Table 1 Schema of the study data collection mapped to study objectives

	Objectives				
Data source	Profile safety climate	Current practice	Falls epidemiology	6-PACK acceptability	Implementation barriers and enablers
Structured observation of patient's bedside		✓			
NUM verbal report			✓		
Audit of medical records, incident reporting and administrative data		✓	✓		
Nurse surveys	✓	✓		✓	✓
Focus groups with nurses		✓	✓	✓	✓
Key informant interviews with senior staff		✓	✓	✓	✓

NUM, nurse unit managers.

Downloaded from http://injuryprevention.bmj.com/ on March 23, 2016 - Published by group.bmj.com

were the same as those detailed for the structured bedside observation and included strategies that were not always possible to observe such as clinical reviews (eg, footwear, medications, mobility and gait aids), toileting regimes and bathroom supervision. Data elements related to fall events were the same as those detailed for the NUM verbal report.

Admitted patients' demographic characteristics and diagnoses were obtained from hospital administrative data sets. Diagnoses were recorded using the International Classification of Diseases, 10th Revision, Australian Modification. Falls data were also obtained by monthly audit of hospital incident reporting and administrative databases. Data elements recorded were the same as those detailed for the NUM verbal report. Radiological investigation reports were reviewed to verify fractures.

Nurse survey

A 43-item survey was developed by the research team following familiarisation with the literature and consultation with the 6-PACK programme developer. Items related to beliefs about falls; current falls prevention practice; 6-PACK programme components; best practice guidelines and key recommendations; and falls reporting practices, and were informed by the TDF (table 2). The survey was combined with the 30-item short version of the Safety Attitudes Questionnaire to measure safety climate.[25] The survey was piloted at the hospital that developed and implemented the 6-PACK programme as part of usual care. The length of the survey was the main issue raised by pilot participants, however, it was deemed difficult to further reduce items without losing important content.

The survey was administered to all eligible nurses over a fortnight period at each site in the preimplementation period. A member of the research team attended handover sessions or designated ward meetings to distribute the survey. During these sessions the researcher described the purpose of the survey, provided the explanatory statement, discussed privacy issues and provided instructions for survey completion. Nurses who volunteered to participate, placed the completed surveys into a sealed box that was collected by the researcher at the end of the 2 week dissemination period. NUMs were not involved in the distribution or administration of the surveys to avoid hierarchical coercion and to ensure strict confidentiality and anonymity of the nurses wishing to participate. No reminders were

Table 2 Mapping of survey, focus group and interview questions to the Theoretical Domain Framework

Survey	Focus group	Interview	Themes	Current practice	Acceptability	Barriers and enablers
Knowledge						
✓	✓	✓	Familiarity with falls prevention best practice guidelines and key recommendations	✓		
	✓	✓	Familiarity with the six interventions included in the 6-PACK programme		✓	
✓	✓	✓	Knowledge of the problem of falls or falls related injuries	✓		
Skills						
	✓	✓	Training required for falls prevention			✓
✓	✓	✓	Ability to undertake falls prevention strategies (risk assessments, implement interventions)	✓		
Memory, attention and decision processes						
	✓	✓	Falls prevention is part of daily care processes	✓		
Behavioural regulation						
	✓	✓	Steps needed to enhance implementation of the 6-PACK programme			✓
✓	✓	✓	Use of audits, reminders and feedback	✓		✓
Beliefs about capabilities						
	✓	✓	Anticipated challenges in implementing the 6-PACK programme at hospital/on ward			✓
Social/professional role and identity						
✓	✓	✓	Role in falls prevention	✓	✓	✓
Emotion						
	✓	✓	The emotional impact of patient falls			✓
Beliefs about consequences						
		✓	Expected outcomes from the 6-PACK programme and how these will be measured		✓	
	✓	✓	Benefits of implementing the 6-PACK programme		✓	
Motivation and goals (Intention)						
	✓	✓	Importance of falls prevention on the ward and how it fits with other patient safety priorities		✓	✓
	✓	✓	Strategies to change falls prevention practice (education, leadership, audit, reminders)			✓
Nature of the behaviours						
✓	✓	✓	Current falls prevention practice and perceived usefulness and effectiveness	✓	✓	
Environmental context and resources						
✓	✓	✓	Suitability of 6-PACK for the ward		✓	
	✓	✓	Complementary/inhibitory activities to 6-PACK implementation (resources, system level factors, time constraints, other patient safety activities—current and planned)		✓	✓
Social influences (norms)						
	✓	✓	Critical people to involve in falls prevention and practice change			✓
✓			Leadership and supervision for falls prevention practice	✓		✓

Downloaded from http://injuryprevention.bmj.com/ on March 23, 2016 - Published by group.bmj.com

provided. Records were also kept to track the number of surveys distributed to, and returned from, each ward in order to calculate response rates.

Focus groups and key informant interviews

Two focus groups and four key informant interviews were scheduled at each of the six hospitals to elicit views on falls, current falls prevention practice, acceptability of the 6-PACK programme and barriers and enablers to practice change. Development of the focus group and interview questions was informed by the TDF (table 2).

Eligible nurses were invited to focus groups via the same method as described for the survey. Key informant interviews were conducted with 24 senior staff from participating hospitals. A range of managers were nominated by the Director of Nursing at each hospital and were invited by letter from the research team.

All focus groups and interviews were led by the same researcher (ALB), and were approximately 1 h in duration. Focus group discussions and interviews were recorded and transcribed, and copies of focus group and interview transcripts provided to participants for verification. Staff participation was voluntary.

Data analysis

Descriptive statistics were calculated to summarise survey responses, falls practice and epidemiology data. Results were collated to identify patterns in climate, practice and falls to provide insights into specific practice changes required to implement the 6-PACK programme and how the programme mapped to underlying demand. Analysis of interview and focus group data was continuous with deductive coding applied and emerging themes explored and tested for applicability and consistency. The aim was to understand the staff's beliefs about falls, the 6-PACK programme and the potential factors that may influence the programme implementation. A detailed analysis plan was devised for each of the five studies and is presented in the respective outcome papers.

Outcome of the project

The investigator team reviewed the findings from the five studies and used them to: (1) refine the drafted programme logic; (2) outline a tailored implementation plan; and (3) develop a site implementation manual and a training package for the site clinical leaders responsible for implementing the 6-PACK programme on the intervention wards.

DISCUSSION

Despite the growing body of falls prevention research,[26] little is known about the most effective ways to change falls prevention practices in hospitals. When considering the broader implementation science evidence, there are also gaps in knowledge as to how to implement complex health interventions most effectively and efficiently in a range of care settings. A systematic review of guideline dissemination and implementation strategies that summarises the findings of 235 studies reported that the majority noted some improvement in care with the use of an implementation plan.[27] The review authors concluded that practice change is possible with the use of a well designed implementation plan; and that no single approach is fit for all interventions and settings. The aim of the preimplementation studies outlined in this protocol was to obtain information on the moderators of the effective use of the 6-PACK falls prevention programme to inform the development of a tailored implementation plan. The findings of these studies have the potential to contribute the much-needed knowledge of implementation science in the field of falls prevention in the acute hospital setting.

There are many reasons for failure to demonstrate the effectiveness of complex health interventions such as falls prevention programmes. System, intervention, clinician and consumer factors may all contribute to their breakdown.[28 29] The use of falls prevention strategies in acute wards can potentially be improved by designing implementation strategies that are tailored to identified barriers and enablers to practice change. Several frameworks exist to guide implementation research.[30] We selected TDF to guide the design of the 6-PACK preimplementation studies. The 12 domains included in the TDF provide an extensive framework for identifying potential barriers to change that can then be mapped to a range of potential implementation strategies. Implementation strategies may include provision of change management resources, leadership (partnerships, executive sponsorship and clinical leaders) and resources (equipment, education and training); integration of programme components into the usual work flow; use of reminders; measurement of practice change (adherence to risk assessment and intervention use); and staff feedback about performance and outcomes.

In conclusion, the 6-PACK preimplementation studies were designed to collect information from multiple sources (senior management, nurses, medical records, bedside observations and practice audits) to provide detailed information on the complexity falls and potential moderators of falls prevention practice in the acute hospital setting. It is a strength of the study design that the assessment of barriers and enablers for implementation were based on a theoretical framework. This enabled the development of theoretically informed implementation strategies that were tailored to the local needs of the wards. Findings of each of the five preimplementation studies and the implementation plan developed and applied in the RCT will be reported in future papers.

Acknowledgements The authors thank the Injury Prevention Unit, The Northern Hospital, Northern Health, Melbourne, Australia who developed the 6-PACK programme. The authors also thank Patricia Livingston and Jason Talevski who provided editorial assistance with the manuscript.

Contributors ALB led the conception, design and application for funding of the project in conjunction with CAB and KDH. ALB, RTM, CAB, KDH and FSL participated in the steering committee and project governance. ALB and DRA led the drafting of all sections of this manuscript. All authors reviewed and revised the manuscript critically for important intellectual content and provided approval of the final version to be published. ALB is the guarantor of the manuscript.

Funding This project was funded by the National Health and Medical Research Council (NHMRC), Australia (APP1007627). ALB's salary was funded by a Career Development Fellowship from the NHMRC (APP1067236). RTM's salary was supported by a postgraduate scholarship from the NHMRC (APP1055604).

Competing interests None declared.

Ethics approval Monash University Human Research Ethics Committee—CF11/0229—2011000072 and each of the relevant hospital ethics committees.

Provenance and peer review Not commissioned; internally peer reviewed.

REFERENCES

1 Australian Commission on Safety and Quality in Health Care. *Preventing Falls and Harm from Falls in Older People: Best Practice Guidelines for Australian Hospitals*. Sydney, Australia: Commonwealth of Australia, 2009.
2 National Institute for Health and Care Excellence. Falls: assessment and prevention of falls in older people. 2013. guidance.nice.org.uk/cg161
3 American Geriatrics Society BGS. *AGS/BGS clinical practice guideline: prevention of falls in older persons*. American Geriatrics Society, 2010.
4 Center for Performance Sciences. *Acute care implementation manual*. Maryland, USA: Centre for Performance Sciences, 2006.

Downloaded from http://injuryprevention.bmj.com/ on March 23, 2016 - Published by group.bmj.com

5 The Australian Council on Healthcare Standards. *National Safety and Quality Health Service Standards Program*. NSW, Australia: The Australian Council on Healthcare Standards, 2012.
6 Healey F, Lowe D, Darowski A, *et al*. Falls prevention in hospitals and mental health units: an extended evaluation of the FallSafe quality improvement project. *Age Ageing* 2014;43:484–91.
7 Rosenthal MB. Nonpayment for performance? Medicare's new reimbursement rule. *N Engl J Med* 2007;357:1573–5.
8 Barker A. *An evaluation of the preventing falls and harm from falls in older people best practice guidelines for Australian hospitals*. Melbourne, Australia: Monash University, 2012.
9 Royal College of Physicians. Report of the 2011 inpatient falls pilot audit. London, UK, RCP, 2012.
10 Royal College of Physicians. National audit of inpatient falls: audit report 2015. London, UK, RCP, 2015.
11 National Patient Safety Agency. *Slips, trips and falls data update*. London, UK: 2010. http://www.nrls.nhs.uk/resources/collections/10-for-2010/reducing-harm-from-falls/
12 Latimer N, Dixon S, Drahota AK, *et al*. Cost–utility analysis of a shock-absorbing floor intervention to prevent injuries from falls in hospital wards for older people. *Age Ageing* 2013;42:641–5.
13 Jørgensen TS, Hansen AH, Sahlberg M, *et al*. Nationwide time trends and risk factors for in-hospital falls-related major injuries. *Int J Clin Pract* 2015;69:703–9.
14 Waters TM, Daniels MJ, Bazzoli GJ, *et al*. Effect of Medicare's nonpayment for hospital-acquired conditions: lessons for future policy. *JAMA Intern Med* 2015;175:347–54.
15 Morello RT, Barker AL, Watts JJ, *et al*. The extra resource burden of in-hospital falls: a cost of falls study. *Med J Aust* 2015;203:367–367.
16 Pittet D, Hugonnet S, Harbarth S, *et al*. Effectiveness of a hospital-wide programme to improve compliance with hand hygiene. Infection Control Programme. *Lancet* 2000;356:1307–12.
17 Campbell M, Fitzpatrick R, Haines A, *et al*. Framework for design and evaluation of complex interventions to improve health. *BMJ* 2000;321:694–6.
18 Baker R, Camosso-Stefinovic J, Gillies C, *et al*. Tailored interventions to overcome identified barriers to change: effects on professional practice and health care outcomes. *Cochrane Database Syst Rev* 2010;(3):CD005470.
19 Barker A, Kamar J, Graco M, *et al*. Adding value to the STRATIFY falls risk assessment in acute hospitals. *J Adv Nurs* 2011;67:450–7.
20 Barker A, Brand C, Haines T, *et al*. The 6-PACK programme to decrease fall-related injuries in acute hospitals: protocol for a cluster randomised controlled trial. *Inj Prev* 2011;17:e5.
21 Barker AL, Kamar J, Morton A, *et al*. Bridging the gap between research and practice: review of a targeted hospital inpatient fall prevention programme. *Qual Saf Health Care* 2009;18:467–72.
22 Morello R, Barker A, Zavarsek S, *et al*. The 6-PACK programme to decrease falls and fall-related injuries in acute hospitals: protocol for an economic evaluation alongside a cluster randomised controlled trial. *Inj Prev* 2012;18:e2.
23 Francis JJ, O'Connor D, Curran J. Theories of behaviour change synthesised into a set of theoretical groupings: introducing a thematic series on the theoretical domains framework. *Implement Sci* 2012;7:35.
24 Savaya R, Waysman M. The logic model. *Admin Soc Work* 2008;29:85–103.
25 Sexton JB, Helmreich RL, Neilands TB, *et al*. The Safety Attitudes Questionnaire: psychometric properties, benchmarking data, and emerging research. *BMC Health Serv Res* 2006;6:44.
26 Cameron ID, Gillespie LD, Robertson MC, *et al*. Interventions for preventing falls in older people in care facilities and hospitals. *Cochrane Database Syst Rev* 2012;12:CD005465.
27 Grimshaw JM, Thomas RE, MacLennan G, *et al*. Effectiveness and efficiency of guideline dissemination and implementation strategies. *Health Technol Assess* 2004;8:iii–iv, 1–72.
28 Brand C, Scott I, Greenberg P, *et al*. Chronic disease management: time for consultant physicians to take more leadership in system redesign. *Intern Med J* 2007;37:653–9.
29 Brand C, Cox S. Systems for implementing best practice for a chronic disease: management of osteoarthritis of the hip and knee. *Intern Med J* 2006;36:170–9.
30 Tabak RG, Khoong EC, Chambers DA, *et al*. Bridging research and practice: models for dissemination and implementation research. *Am J Prev Med* 2012;43:337–50.

Downloaded from http://injuryprevention.bmj.com/ on March 23, 2016 - Published by group.bmj.com

IP

Development of an implementation plan for the 6-PACK falls prevention programme as part of a randomised controlled trial: protocol for a series of preimplementation studies

Anna L Barker, Renata T Morello, Darshini R Ayton, Keith D Hill, Fiona S Landgren and Caroline A Brand

Inj Prev published online March 1, 2016

Updated information and services can be found at:
http://injuryprevention.bmj.com/content/early/2016/03/01/injuryprev-2015-041915

These include:

References This article cites 20 articles, 6 of which you can access for free at:
http://injuryprevention.bmj.com/content/early/2016/03/01/injuryprev-2015-041915#BIBL

Email alerting service Receive free email alerts when new articles cite this article. Sign up in the box at the top right corner of the online article.

Topic Collections Articles on similar topics can be found in the following collections

Clinical trials (epidemiology) (114)
Epidemiologic studies (836)

Notes

To request permissions go to:
http://group.bmj.com/group/rights-licensing/permissions

To order reprints go to:
http://journals.bmj.com/cgi/reprintform

To subscribe to BMJ go to:
http://group.bmj.com/subscribe/
